# SARS-CoV-2 Seroprevalence in Employees of Four Essential Non–Health Care Sectors at Moderate/High Risk of Exposure to Coronavirus Infection: Data From the “First Wave”

**DOI:** 10.1097/JOM.0000000000002690

**Published:** 2022-09-09

**Authors:** Giulia Belloni, Julien Dupraz, Audrey Butty, Jérôme Pasquier, Sandrine Estoppey, Murielle Bochud, Semira Gonseth-Nussle, Valérie D'Acremont

**Affiliations:** From the Center for Primary Care and Public Health (Unisanté), University of Lausanne, Lausanne, Switzerland.

**Keywords:** antibodies, COVID-19, serology, workers, workplace

## Abstract

The aim of this study was to evaluate severe acute respiratory syndrome coronavirus 2 (SARS-CoV-2) seroprevalence in Swiss non–health care employees at a moderate to high risk of exposure: bus drivers and supermarket, laundry service, and mail-sorting center employees.

**Methods:**

Data on 455 essential workers included demographics, SARS-CoV-2 exposure and use of protective measures. Anti–SARS-CoV-2 immunoglobulins G and A targeting the spike protein were measured between May and July 2020.

**Results:**

The overall crude seroprevalence estimate (15.9%; 95% confidence interval [CI], 12.6% to 19.7%) among essential workers was not significantly higher than that of the general working-age population (11.2%; 95% CI, 7.1% to 15.2%). Seroprevalence ranged from 11.9% (95% CI, 6.3% to 19.8%) among bus drivers to 22.0% (95% CI, 12.6% to 19.7%) among food supermarket employees.

**Conclusions:**

We found no significant difference in seroprevalence between our sample of essential workers and local working-age population during the first lockdown phase of the COVID-19 pandemic. Having a seropositive housemate was the strongest predictor of SARS-CoV-2 seropositivity.

## Abstract 2

Our results indicate the importance of combining work-specific protective measures with public health policies in the private settings: at-home exposure might have been the most probable source of infections among these essential employees and the implementation of protective measures against SARS-CoV-2 infection at workplace may have mitigated their risk of occupational exposure.

**CME Learning Objectives**

After completing this enduring educational activity, the learner will be better able to:

- Evaluate SARS-CoV-2 seroprevalence in Swiss non-healthcare employees at a moderate to high risk of exposure
- Discuss the difference in SARS-CoV-2 seroprevalence between essential workers of four sectors and general working-age population
- Identify characteristics associated with SARS-CoV-2 seropositivity among these essential workers

In response to the serious global health hazard posed by the severe acute respiratory syndrome coronavirus 2 (SARS-CoV-2), the World Health Organization (WHO) declared the SARS-CoV-2 outbreak a global pandemic on March 11, 2020.^1^ In Switzerland, the first coronavirus disease 2019 (COVID-19) case was registered on February 25, 2020,^2^ and the first COVID-19 wave occurred in late March and ended by late May.^3,4^ During this period, Swiss authorities adopted a wide range of lockdown protective measures in many sectors (ie, health, economy, mobility, employment) to contain SARS-CoV-2's rapid spread, protect citizens, and mitigate the economic burden of the pandemic. Among work-health policies, all nonessential businesses and activities were closed, and remote work was recommended whenever possible to reduce workplace infections. However, some essential work could neither be discontinued nor done at home, which placed employees at greater risk of exposure to SARS-CoV-2.^5,6^ Essential workers are defined as those conducting a range of operations and services in facilities that are indispensable to preserving life, health, and basic societal functioning. Essential workers include health care workers and employees of all critical infrastructures.^7–9^

The assessment of SARS-CoV-2 seroprevalence among different types of essential workers is important to provide relevant information on the real work-related risk of exposure, to support the development of protective measures for employees, to reduce the operational impact, and to evaluate policies effectiveness.^10–12^ Indeed, assessing the presence of circulating SARS-CoV-2 antibodies can be used to estimate exposure to the virus, thanks to the ability to identify past infections, including asymptomatic forms.^13,14^

Two population-based studies, carried out in Iran and in Geneva, Switzerland, evaluated SARS-CoV-2 seroprevalence among non–health care essential workers during the first national lockdown. Although the magnitude of seropositivity variation across work sectors differed in the two studies, both found an overall seroprevalence similar to that of the general population.^10,15^ With regard to specific occupations, Stringhini et al^15^ observed the highest proportion of seropositive workers among kitchen staff of nursing homes. Findings among first responders are inconsistent: two studies found SARS-CoV-2 seroprevalence in police and firefighters close to that of the general population.^16,17^ Conversely, Brazilian military police^18^ and US law enforcement and firefighters had higher seroprevalence than that of the general population.^19^ Moreover, Sami et al^16^ detected a twice-higher seroprevalence among correctional staff and emergency medical technicians compared with the general population.

The very high seroprevalence of 50.3% (compared with 34% in the community) found in the staff of pharmaceutical and hardware companies in Karachi (Pakistan) was attributed to the delay in the implementation of lockdown measure.^20^

Further research about SARS-CoV-2 seroprevalence among essential workers is needed. First, there is a lack of serological studies in high-density workplaces that were affected by SARS-CoV-2 outbreaks, such as meat processing facilities and call centers.^21–24^ Second, in contrast to the extensive research on SARS-CoV-2 infections in health care employees,^25,26^ only few research studies evaluated SARS-CoV-2 seroprevalence among other categories of workers. Third, in absence of data from a comparison group, such as the general population or workers experiencing a low-risk of exposure,^11,27–29^ it is difficult to assess the magnitude of the risk among highly exposed employees.

Assessing SARS-CoV-2 seroprevalence in employees at different occupational risk of exposure, while taking into account the precaution measures applied in the workplace and in private life, can help public health authorities and employers to better target and tailor protective interventions.

This study aimed to evaluate SARS-CoV-2 seroprevalence (immunoglobulin G [IgG] and/or IgA) in employees of four non–health care critical infrastructures. We postulated a moderate to high risk of SARS-CoV-2 occupational exposure for these workers when at least one of the following criteria was met: difficulty/impossibility to work from home, physical proximity with colleagues or customers, overcrowded workplaces, and handling or being in contact with potentially infectious material.

We hypothesized two scenarios: (1) a higher SARS-CoV-2 seroprevalence than that in the general population of the same age due to a higher exposure to the virus and (2) a seroprevalence similar to that of the general population due to similar exposure or higher exposure but proper implementation of protective measures in the workplace.

## METHODS

### Study Design and Population

This cross-sectional study was conducted among workers of four companies operating in essential sectors in the Canton of Vaud, Switzerland. Study participants were bus drivers of a public transport company and employees of the following workplaces: five stores of a food supermarket company, a mail-sorting center of a postal service, and four sites of a laundry operating in the health care sector. These workplaces are at moderate to high risk of SARS-CoV-2 exposure according to WHO.^30^ Notably, WHO classification defined a “medium exposure risk” for jobs/tasks with close, frequent contact with the general public and a “high exposure risk” for jobs/tasks with close contact with people more likely to have COVID-19, as well as contact with objects and surfaces possibly contaminated with the virus.^30^

**FIGURE 1:**
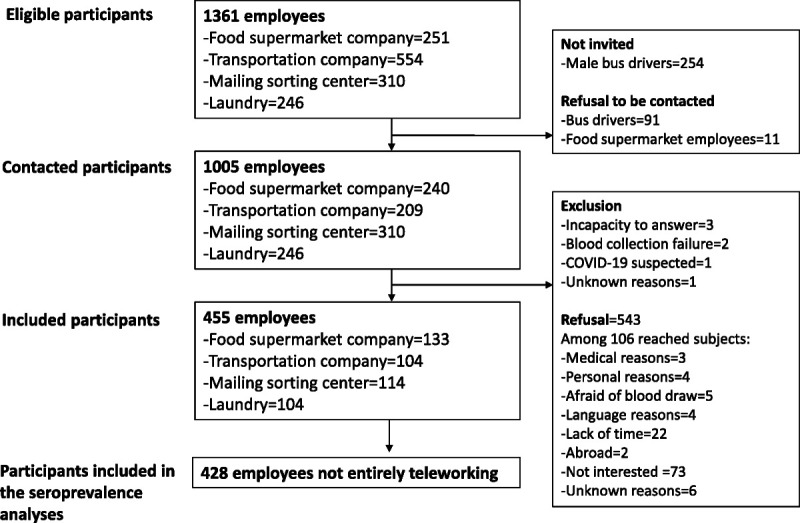
Flowchart of participants.

Overall, 1361 employees, partially or fully on duty from March 1 to April 30, 2020, were eligible to participate (Fig. 1).

Taking into account the high number of eligible bus drivers (n = 554) compared with the other types of employees, a subsample of 300 bus drivers was invited to participate by taking all women (n = 44) and a random sample of 256 men. Because 91 bus drivers and 11 food supermarket workers refused to be contacted, 1005 participants were invited to participate by postal mailing and informed of the study goals and design. Overall, 455 participants, who had provided informed consent, completed a self-administered online questionnaire, and provided a blood specimen for the detection of antibodies against SARS-CoV-2, were included in the study (Fig. 1). The questionnaire assessed participants' demographics, SARS-CoV-2 exposure, and protection behaviors used in private life and at the workplace. Blood sample collection was done at study sites by trained health care staff, from May 25 to July 7, 2020. Part of blood samples was directly analyzed at Lausanne University Hospital's laboratory, and the remaining specimens were aliquoted and stored in the study center's biobank (Unisanté's biobank). We excluded 27 employees who reported entirely working from home from the seroprevalence analyses because they were not at a higher risk of occupational SARS-CoV-2 exposure than the general population. The final study population of 428 employees was “participants working on site” (Fig. 1). The participation rate was 45.3%, and reasons for exclusion/refusal are listed in Figure 1. Moreover, each company provided the list of the protective measures implemented against SARS-CoV-2 infection in their workplaces during the study period (Table 1). This study received approval by the cantonal ethics committee of Vaud (protocol CER-VD 2020-00887) on April 23, 2020.

**TABLE 1:** List of the Protective Measures Against SARS-CoV-2 Infection Implemented at Workplace by Each Company

	Food Supermarket	Laundry Operating in Health Care Sector	Postal Service	Public Transportation
Date of introduction	March 20, 2020	Beginning of March 2020	Mid-March 2020	Beginning of March 2020
Hand disinfection	✓	✓	✓	✓
Social distancing in common areas	✓	✓	✓	✓
(Floor) markings for social distancing	✓	✓		✓
Increased common areas/equipment cleaning	✓	✓	✓	✓
Info on the virus and protective measures (ie, posters, FAQ of public health authority, informative e-mails)	✓	✓		✓
Face mask availability		✓	✓	✓
Use of face masks if social distancing could not be respected and for those at higher risk		✓		✓
Cafeterias adaptation		✓		✓
Staggered entry		✓		✓
Other measures	Count of the number of people in the market Personal hand-sanitizer distribution Social distancing audio reminders Plexiglas for supermarket checkout points	Implementation of virtual meeting Vulnerable employees' suspension (early June) Display of correct handwashing Temperature screening on entry Information on implemented measures at the workplace and on the correct use of the mask Stages and visit suspension Wear a gown (for sorting sector) Safety glass availability	Staggered breaks Interruption of staff pairing at the workplace Reusable cup ban External persons are not allowed to use the restaurant Cancellation of team briefing Door adaptation to be opened with the arm Keep open all doors in common areas Installation of outdoor toilet for external people Stages suspension Smokers' booth closure Work from home, if possible and office reorganization to keep social distance	Personal hand-sanitizer distribution Physical barriers between workers and customers Bus front door closure Temporary suspension of ticket sales on the bus Temporary suspension of minibus

SARS-CoV-2, severe acute respiratory syndrome coronavirus 2.

### SARS-CoV-2 Antibodies Detection

Anti–SARS-CoV-2 antibodies targeting the spike (S) protein in its native trimeric form were measured using a Luminex immunoassay developed by the Lausanne University Hospital (Lausanne University Hospital), in collaboration with the École Polytechnique Fédérale de Lausanne.^31^ Specificity of the Luminex S protein trimer assay was 99.2% for IgG (in sera from people infected with prepandemic coronaviruses or from patients with autoimmune diseases) and 98.5% for IgA (in sera from pre–COVID-19 healthy adults). Sensitivity estimate for IgG and IgA, using sera from patients with recently documented COVID-19, was 42.1% and 68.8% at 6 to 10 days after symptoms appeared, 91.7% and 94.4% at 10 to 15 days, and 96.6% and 90% at 16 to 33 days, respectively.^31^ We defined the threshold for a positive result at an antibody multiplex fluorescent immunoassay ratio of ≥6 for IgG and ≥6.5 for IgA. In our study, the SARS-CoV-2 seropositivity was defined as positivity on at least one of the two tests.

### Covariates

Data collected by self-completed questionnaires related to demographics, medical history, and SARS-CoV-2 exposure via housemates. Personal SARS-CoV-2 exposure and protective behaviors in private life, changes in work conditions, and implementation of home-based work were evaluated. Data about exposure and protective behaviors in the workplace were obtained exclusively from participants working on site. We defined a contact for more than 15 minutes within 1.5 m as a close contact. The semilockdown referred to the period from March 16 to May 10, 2020.

### Statistical Analyses

Participants' characteristics for each company were analyzed with descriptive statistics. Crude seroprevalence was calculated as a proportion with 95% confidence intervals (CIs) for all participants working on site and for subgroups defined according to their work sector, workplace or work function. We combined participants from the three smallest workplaces of the supermarket company because of the similar settings and the limited number of participants per workplace. Similarly, we combined the postal employees working together in a great hall because of a similar occupational risk. Because all food supermarket workers were in contact with customers, we did not perform stratified analyses by work function. We calculated the difference in seroprevalence between essential workers and the general population aged 20 to 64 years during the same period and the corresponding 95% CI and assessed statistical significance using the *χ*^2^ test or, when appropriate, the Fisher exact test. General working-age population (20 to 64 years old) came from a sample (n = 235) of noninstitutionalized residents randomly selected from the population registry of the Canton of Vaud. We compared the characteristics of participants working on site according to their SARS-CoV-2 serology result using Student *t* test for continuous variables and *χ*^2^ test for categorical variables; if expected counts were less than 5, we applied the Fisher exact test. We performed a multivariable logistic regression to evaluate characteristics associated with SARS-CoV-2 seropositivity. Adjustment variables were age, sex, work sector, and all variables significantly associated in bivariable analyses, adopting a significance level of 0.1, with the exception of variables issued from the same branching logic, for which only the most pertinent was kept.

Missing data were excluded from the analyses. All statistical analyses were conducted using Stata version 15.0 (StataCorp LLC, College Station, TX). Statistical significance was set at a level of *P* < 0.05.

## RESULTS

Characteristics of the 455 essential workers included in the study are listed according to the work sector in Table A as Supplemental Digital Content, http://links.lww.com/JOM/B191. The average age was 44.3 (SD, 11.5) years, with the oldest group consisting of the bus drivers (47.1 [SD, 9.3] years). Female workers were predominant in food supermarkets (65.4%) and in the laundry services (77.9%), whereas only 16 bus drivers (15.4%) were women. Laundry employees adopted more protective behaviors against SARS-CoV-2, both in private life and at the workplace, than the other types of workers. For example, laundry employees implemented a higher use of masks in public places (33.7% vs 5.8% among bus drivers, 3.0% in food supermarket employees, and 7.9% among mail-sorting center workers) and at work (83.8% vs 4.0% among bus drivers, 3.3% in food supermarket employees, and 1.9% among mail-sorting center workers). Food supermarket workers reported more frequent close contact at the workplace, with people having symptoms suggestive of SARS-CoV-2 infection (22.8% vs 14% in all employees) or with persons having tested positive for SARS-CoV-2 (17.1% vs 7.2%) (Table A as Supplemental Digital Content, http://links.lww.com/JOM/B191).

Among the 428 participants working on site, 68 tested positive for SARS-CoV-2 IgG and/or IgA (Table 2), and among them, 37 (54.4%) reported flulike symptoms since the end of February 2020. The overall crude SARS-CoV-2 seroprevalence was 15.9% (95% CI, 12.6% to 19.7%) (Table 2). The seroprevalence found in the local general population aged 20 to 64 years at the same period was 11.2% (7.1% to 15.2%). The seroprevalence in the selected essential workers was thus higher (risk ratio, 1.44, 0.94 to 2.19) than that in the general population, but not significantly higher (*P* = 0.089). When each work sector was considered independently, only food supermarket workers had a statistically significant higher seroprevalence (22.0%, 15.0% to 30.3%, *P* = 0.006). Notably, compared with the general population, the seroprevalence of workers in store 5 was more than three times higher (37.9%, 25.5% to 51.6%, *P* < 0.001). Regarding the other sectors, the seroprevalence was highest among employees of the mail-sorting service (16.2%, 9.7% to 24.7%), followed by the laundry services' employees (12.1%, 6.4% to 20.2%) and the bus drivers (11.9%, 6.3% to 19.8%).

**TABLE 2:** SARS-CoV-2 Seroprevalence Among Participants Working on Site, Overall, and According to Their Work Sector, Workplace, and Work Function

	SARS-CoV-2 Positivity		Seroprevalence	Difference With the Local General Population	P*
	No	Yes	(95% CI)	(95% CI)	
All sectors (n = 428)	360	68	15.9 (12.6–19.7)	4.7 (−0.6 to 10.0)	0.089
Food supermarket (n = 123)	96	27	22.0 (15.0–30.3)	10.8 (2.4 to 19.1)	0.006
Stores 1–3	17	0	0 (0–19.5)	—	
Store 4	43	5	10.4 (3.5–22.7)	−0.7 (−10.3 to 8.8)	0.896
Store 5	36	22	37.9 (25.5–51.6)	26.8 (13.6 to 39.9)	<0.001
Public transportation (n = 101)	89	12	11.9 (6.3–19.8)	0.7 (−6.7 to 8.2)	0.828
Mail-sorting service (n = 105)	88	17	16.2 (9.7–24.7)	5.0 (−3.1 to 13.1)	0.189
Office managers	12	0	0 (0–26.5)	—	
Employees who worked in the large hall^†^	76	17	18.3 (11.0–27.6)	7.1 (−1.7 to 15.9)	0.081
Laundry (n = 99)	87	12	12.1 (6.4–20.2)	1.0 (−6.6 to 8.5)	0.781
Cleaning of health workers' professional clothes (two laundry sites)	42	7	14.3 (5.9–27.2)	3.1 (−7.5 to 13.7)	0.522
Cleaning of long-term residents' private clothes (one laundry site)	14	0	0 (0–23.2)	—	
Managers and administrative staff	15	4	21.1 (6.1–45.6)	9.9 (−8.9 to 28.7)	0.244
Technician	8	0	0 (0–36.9)	—	
Cleaning of clothes at the health care facilities (onsite)	8	1	11.1 (0.3–48.2)	−0.1 (−21.0 to 20.9)	1.000

Seroprevalence among general population (n = 235) aged 20 to 64 years = 11.2% (7.1%–15.2%).

*From *χ*^2^ test or Fisher exact test.

^†^Workers employed in the following activities: sorting, production, support, and technicians.

CI, confidence interval; SARS-CoV-2, severe acute respiratory syndrome coronavirus 2.

No other statistically significant difference with the local general population aged 20 to 64 years was found stratifying by work function or workplace, including the four laundry workplaces (Table 2). The participants' characteristics according to serology result are displayed in Table B as Supplemental Digital Content, http://links.lww.com/JOM/B192. In multivariable analysis, variables associated with seropositivity were being a food supermarket worker (adjusted odds ratio [aOR], 2.67; 95% CI, 1.01 to 7.10; *P* = 0.049), having experienced flulike symptoms since the end of February 2020 (aOR, 2.65; 95% CI, 1.43 to 4.88; *P* = 0.002), having at least one housemate who tested positive by reverse transcriptase–polymerase chain reaction (aOR, 8.16; 95% CI, 1.41 to 47.08; *P* = 0.019), and respecting hygiene rules in private life (aOR, 3.81; 95% CI, 1.09 to 13.39; *P* = 0.037) (Table 3).

**TABLE 3:** Multivariable Comparison of Characteristics of Participants Working on Site According to SARS-CoV-2 Seropositivity (n = 411)

	OR (95% CI)	P*
Age, y	1.02 (1.00–1.05)	0.107
Women	0.86 (0.44–1.71)	0.673
Company		
Public transportation	Reference	
Food supermarket	2.67 (1.01–7.10)	0.049
Mail-sorting service	1.63 (0.64–4.16)	0.308
Laundry	1.28 (0.44–3.78)	0.651
Flulike symptoms since the end of February 2020	2.65 (1.43–4.88)	0.002
Having at least one housemate tested RT-PCR positive	8.16 (1.41–47.08)	0.019
At least one close contact with people, other than the housemates, having symptoms suggestive of COVID-19,^†^ from 24 h before symptom onset	1.14 (0.47–2.77)	0.765
Respect of hygiene rules in private life^‡^	3.81 (1.09–13.39)	0.037
Wearing always a mask in public places	2.49 (0.99–6.31)	0.054
Change in one's working conditions since SARS-CoV-2	1.55 (0.84–2.88)	0.163
At least one close contact at work with a person tested positive for SARS-CoV-2, from 24 h before the outbreak of symptoms	2.32 (0.83–6.49)	0.110

Adjusted model includes all variables listed.

*From multivariable logistic regression.

^†^Defined as presenting cough or sore throat or shortness of breath or fever or fatigue or muscle pain or loss of smell or taste.

^‡^Defined as frequent handwashing, sneezing into the elbow, use of disposable handkerchiefs, and so on.

CI, confidence interval; OR, odds ratio; RT-PCR, reverse transcriptase–polymerase chain reaction; SARS-CoV-2, severe acute respiratory syndrome coronavirus 2.

## DISCUSSION

This study investigated the SARS-CoV-2 seroprevalence during the first semilockdown in the Canton of Vaud, Switzerland, among four types of essential workers who are at theoretical moderate to high risk of exposure due to their close interactions with customers or colleagues and/or contact with potentially infected surfaces/material. The SARS-CoV-2 seroprevalence was overall higher (but not significantly) than that of local general working-age population. However, the seroprevalence varied according to the work sector, being similar among bus drivers and workers at the post service and at the laundries, but higher among food supermarket employees, compared with that of the general population. A history of flulike symptoms, having a housemate with a confirmed COVID-19 infection, compliance with hygiene rules in private life, and being a food supermarket worker significantly increased the odds of testing positive for SARS-CoV-2 antibodies.

Consistent with previous findings,^10,15^ the overall risk of SARS-CoV-2 infection among these four types of essential workers was not significantly increased. These findings might be the result of the appropriate implementation of safety procedures at the workplace. Two facts are in favor of this hypothesis. First, all four companies put in place, during the study period, a range of measures to protect their employees and customers (Table 1). Second, a lower seroprevalence was found among employees of the laundry, who better implemented protective measures, whereas the highest seropositivity was detected among food supermarket workers who reported a less adequate implementation of protective measures at work. Furthermore, the low seroprevalence among bus drivers could be partially attributed to the decreased use of public transport during the first wave.

Another hypothesis is that most workers got infected at home rather than at work and therefore had the same risk of exposure as the general working-age population. Indeed, consistent with results of previous research among essential workers,^16,32^ having a housemate who tested positive for SARS-CoV-2 was associated with seropositivity in our study. Conversely, after controlling for potential confounding variables, having a close workplace contact with a person who tested positive for SARS-CoV-2 was not associated with seropositivity. This is consistent with similar results among health care workers showing that SARS-CoV-2 seroconversion was associated with household transmission, but not with working in a COVID-19 unit.^32,33^ Moreover, previous research found a higher risk of infection from exposure to a household member than from other types of exposure.^34,35^

A higher seroprevalence than that of the general working-age population was found for food supermarket workers. Specifically, this was the case for one of the five food stores, suggesting that exposure to SARS-CoV-2 may have occurred mostly in the workplace rather than at home for employees of this specific store (store 5). Taking into account that all food stores implemented the same kind of precautionary measures, we hypothesized two scenarios: (1) an outbreak occurred within store 5 through contacts among colleagues, probably when they were eating close to each other and without masks during breaks; (2) an exposure to customers with a much higher incidence of COVID-19 than customers at other stores, which is supported by a high number of COVID-19 cases in the city of store 5 during the first weeks of the epidemic (oral communication from the Office of the Chief Medical Officer of Canton of Vaud to V.D.A.). One could argue that the latter observation would support the hypothesis of workers having been infected at home rather than at work; however, many food workers of store 5 were, in fact, living in other cities with lower transmission. Further detailed analyses in this subsample could not be performed because of the small number of store 5 participants (n = 58).

Regarding mail-sorting employees, the overall seroprevalence was higher than that of the general working-age population but not significantly. Interestingly, nearly a quarter of the people working in the main hall were positive, but no cases were found among office staff. This suggests that transmission within a large open space, where close contacts with many different collaborators cannot be avoided, might still have played a role. The finding that complying with hygiene rules in private life (ie, frequent handwashing, sneezing into the elbow, using disposable handkerchiefs) was associated with seropositivity was unexpected. It might be a chance finding, because an elevated compliance with the hygiene rules was observed in most participants (94.1% of seropositive vs 82.8% of seronegative workers). Finally, our finding that reporting a history of flulike symptoms was associated with SARS-CoV-2 seropositivity is in line with other studies among essential workers.^10,36^

This study has some limitations. First, the participation rate was lower than expected, limiting the precision of the results. Second, participants with a history of COVID-19–like symptoms, as well as those with a household or a work exposure, were more likely to take part in the study, potentially leading to overestimating the seroprevalence. However, because this applies also to the local working-age population, it should not impact the difference in seroprevalence. Finally, the local general population sample aged 20 to 64 years was small (n = 235) and may include other essential workers at moderate to high occupational risk of exposure to coronavirus infection, which may lead to an underestimation of the difference in SARS-COV-2 seroprevalence between the study population and this comparison group.

One clear strength of this study is the extensive set of covariates collected and taken into account as potential confounders in multivariable analysis.

## CONCLUSIONS

The overall SARS-CoV-2 seroprevalence among essential workers of four sectors was similar to that of the general working-age population during the first COVID-19 wave in the Canton of Vaud. The implementation of protective measures against SARS-CoV-2 infection at the workplace could have mitigated the risk of exposure. Our results show that exposure in the workplace may have contributed to transmission among food-store workers. However, as shown in several studies looking at other types of workers, “at home” exposure seems to be the most probable source of infections among these essential workers, which is also suggested by the fact that the strongest predictor of seropositivity was having a housemate positive for SARS-CoV-2. Our results highlight the importance of combining work-specific protective measures with universal public health measures to be applied in private settings.
