# Supplementary material for: SARS-CoV-2 Seroprevalence in Employees of Four Essential Non–Health Care Sectors at Moderate/High Risk of Exposure to Coronavirus Infection: Data From the “First Wave”
Source: J Occup Environ Med. 2022 Sep 9;65(1):10–5. doi: 10.1097/JOM.0000000000002690 (PMC9835238; doi:10.1097/JOM.0000000000002690)
Supplement: Supplementary file 1 [file joem-65-010-s001.docx]

**ABSTRACT**

**Objective:** to evaluate SARS-CoV-2 seroprevalence in Swiss non-healthcare employees at a moderate to high risk of exposure: bus drivers; supermarket, laundry service, and mail-sorting center employees.

**Methods**: Data on 455 essential workers included demographics, SARS-CoV-2 exposure and use of protective measures. Anti-SARS-CoV-2 IgG and IgA targeting the spike protein were measured between May and July 2020.

**Results:** The overall crude seroprevalence estimate (15.9%, 95% CI=12.6-19.7) among essential workers was not significantly higher than that of the general working-age population (11.2%, 95% CI=7.1-15.2). Seroprevalence ranged from 11.9% (95% CI=6.3-19.8) among bus drivers to 22.0% (95% CI=12.6-19.7) among food supermarket employees.

**Conclusions**: We found no significant difference in seroprevalence between our sample of essential workers and local working-age population during the first lockdown phase of the COVID-19 pandemic. Having a seropositive housemate was the strongest predictor of SARS-CoV-2 seropositivity.

**Keywords:** COVID-19, serology, antibodies, workplace, workers

**INTRODUCTION**

In response to the serious global health hazard posed by the severe acute respiratory syndrome coronavirus 2 (SARS-CoV-2), the World Health Organization (WHO) declared the SARS-CoV-2 outbreak a global pandemic on March 11, 2020.(1). In Switzerland, the first coronavirus disease 2019 (COVID-19) case was registered on February 25, 2020 (2) and the first COVID-19 wave occurred in late March and ended by late May (3, 4). During this period, Swiss authorities adopted a wide range of lockdown protective measures in many sectors (i.e. health, economy, mobility, employment) to contain SARS-CoV-2’s rapid spread, protect citizens and mitigate the economic burden of the pandemic. Among work-health policies, all non-essential businesses and activities were closed and remote work was recommended whenever possible in order to reduce workplace infections. However, some essential work could neither be discontinued nor done at home, which placed employees at greater risk of exposure to SARS-CoV-2 (5, 6). Essential workers are defined as those conducting a range of operations and services in facilities which are indispensable to preserving life, health and basic societal functioning. Essential workers include healthcare workers and employees of all critical infrastructures (7-9).

The assessment of SARS-CoV-2 seroprevalence among different types of essential workers is important to provide relevant information on the real work-related risk of exposure, to support the development of protective measures for employees, to reduce the operational impact and to evaluate policies effectiveness (10-12). Indeed, assessing the presence of circulating SARS-CoV-2 antibodies can be used to estimate exposure to the virus, thanks to the ability to identify past infections, including asymptomatic forms (13, 14).

Two population-based studies, carried out in Iran and in Geneva, Switzerland, evaluated SARS-CoV-2 seroprevalence among non-healthcare essential workers during the first national lockdown. Although the magnitude of seropositivity variation across work sectors differed in the two studies, both found an overall seroprevalence similar to that of the general population (10, 15). With regards to specific occupations, Stringhini et al. observed the highest proportion of seropositive workers among kitchen staff of nursing homes (15). Findings among first responders are inconsistent: two studies found SARS-CoV-2 seroprevalence in police and firefighters close to that of the general population (16, 17). Conversely, Brazilian military police (18) and US law enforcement and firefighters had higher seroprevalence than that of the general population (19). Moreover, Sami et al. detected a twice-higher seroprevalence among correctional staff and emergency medical technicians compared to the general population (16).

The very high seroprevalence of 50.3% (compared to 34% in the community) found in the staff of pharmaceutical and hardware companies in Karachi (Pakistan) was attributed to the delay in the implementation of lockdown measure (20).

Further research about SARS-CoV-2 seroprevalence among essential workers is needed. First, there is a lack of serological studies in high-density workplaces that were affected by SARS-CoV-2 outbreaks, such as meat processing facilities and call centers (21-24). Second, in contrast to the extensive research on SARS-CoV-2 infections in health-care employees (25, 26), only few research studies evaluated SARS-CoV-2 seroprevalence among other categories of workers. Third, in absence of data from a comparison group, such as the general population or workers experiencing a low-risk of exposure (11, 27-29), it is difficult to assess the magnitude of the risk among highly exposed employees.

Assessing SARS-CoV-2 seroprevalence in employees at different occupational risk of exposure, while taking into account the precaution measures applied in the workplace and in private life, can help public health authorities and employers to better target and tailor protective interventions.

This study aimed to evaluate SARS-CoV-2 seroprevalence (IgG and/or IgA) in employees of four non-healthcare critical infrastructures. We postulated a moderate-to-high risk of SARS-CoV-2 occupational exposure for these workers when at least one of the following criteria was met: difficulty/impossibility to work from home, physical proximity with colleagues or customers, overcrowded workplaces, and handling or being in contact with potentially infectious material.

We hypothesized two scenarios: 1) a higher SARS-CoV-2 seroprevalence than that in the general population of the same age due to a higher exposure to the virus; 2) a seroprevalence similar to that of the general population due to similar exposure or higher exposure, but proper implementation of protective measures in the workplace.

**METHODS**

**Study design and population**

This cross-sectional study was conducted among workers of four companies operating in essential sectors in the Canton of Vaud, Switzerland. Study participants were bus-drivers of a public transport company and employees of the following workplaces: five stores of a food supermarket company, a mail-sorting center of a postal service and four sites of a laundry operating in the healthcare sector. These workplaces are at moderate to high risk of SARS-CoV-2 exposure according to WHO (30). Notably, WHO classification defined a “*medium exposure risk”* for jobs/tasks with close, frequent contact with the general public and a “*high exposure risk”* for jobs/tasks with close contact with people more likely to have COVID-19, as well as contact with objects and surfaces possibly contaminated with the virus (30).

Overall, 1361 employees, partially or fully on duty from March 1^st^ to April 30^th^ 2020, were eligible to participate (Figure 1).

**Figure 1. Flow chart of participants**

Taking into account the high number of eligible bus drivers (n=554) compared to the other types of employees, a subsample of 300 bus drivers was invited to participate by taking all women (n=44) and a random sample of 256 men. Because 91 bus drivers and 11 food supermarket workers refused to be contacted, 1005 participants were invited to participate by postal mailing, and informed of the study goals and design. Overall, 455 participants, who had provided informed consent, completed a self-administered online questionnaire and provided a blood specimen for the detection of antibodies against SARS-CoV-2, were included in the study (Figure 1). The questionnaire assessed participants’ demographics, SARS-CoV-2 exposure, and protection behaviors used in private life and at the workplace. Blood sample collection was done at study sites by trained health care staff, from 25^th^ May to 7^th^ July, 2020. Part of blood samples were directly analyzed at the Lausanne University Hospital’s (CHUV) laboratory and the remaining specimens were aliquoted and stored in the study center’s biobank (Unisanté’s biobank). We excluded 27 employees who reported entirely working from home from the seroprevalence analyses because they were not at a higher risk of occupational SARS-CoV-2 exposure than the general population. The final study population of 428 employees were “participants working on site” (Figure 1). The participation rate was 45.3%, and reasons for exclusion/refusal are listed in Figure 1. Moreover, each company provided the list of the protective measures implemented against SARS-CoV-2 infection in their workplaces during the study period (Table 1). This study received approval by the cantonal ethics committee of Vaud (protocol CER-VD 2020-00887) on 23^rd^ of April, 2020.

**SARS-CoV-2 antibodies detection**

Anti-SARS-CoV-2 antibodies targeting the spike (S) protein in its native trimeric form were measured using a Luminex immunoassay developed by the Lausanne University Hospital (CHUV, Switzerland), in collaboration with the École Polytechnique Fédérale de Lausanne (EPFL)(31). Specificity of the Luminex S protein trimer assay was 99.2% for IgG (in sera from people infected with pre-pandemic coronaviruses or from patients with autoimmune diseases) and 98.5% for IgA (in sera from pre-COVID-19 healthy adults). Sensitivity estimate for IgG and IgA, using sera from patients with recently documented COVID-19, was 42.1% and 68.8% at 6 to 10 days post-symptoms, 91.7% and 94.4% at 11 to 10 days, and 96.6% and 90% at 16 to 33 days, respectively (31). We defined the threshold for a positive result at an antibody Multiplex Fluorescent Immunoassay (MFI) ratio of ≥6 for IgG and ≥6.5 for IgA. In our study, the SARS-CoV-2 seropositivity was defined as positivity on at least one of the two tests.

**Covariates**

Data collected by self-completed questionnaires related to demographics, medical history and SARS-CoV-2 exposure via housemates. Personal SARS-CoV-2 exposure and protective behaviors in private life, changes in work conditions and implementation of home-based work were evaluated.. Data about exposure and protective behaviors in the workplace were obtained exclusively from participants working on site. We defined a contact for more than 15 minutes within 1.5 meter as a close contact. The semi-lockdown referred to the period from March 16 to May 10, 2020.

**Statistical analyses**

Participants’ characteristics for each company were analyzed with descriptive statistics. Crude seroprevalence was calculated as a proportion with 95% confidence intervals (CI) for all participants working on site and for subgroups defined according to their work sector, workplace or work function. We combined participants from the three smallest workplaces of the supermarket company due to the similar settings and the limited number of participants per workplace. Similarly, we combined the postal employees working together in a great hall because of a similar occupational risk. Since all food supermarket workers were in contact with customers, we did not perform stratified analyses by work function. We calculated the difference in seroprevalence between essential workers and the general population aged 20-64 years old during the same period and the corresponding 95% confidence interval (CI) and assessed statistical significance using the chi-squared test or, when appropriate, the Fisher’s exact test. General working-age population (20-64 years old) came from a sample (n=235) of non-institutionalized residents randomly selected from the population registry of the canton of Vaud. We compared the characteristics of participants working on site according to their SARS-CoV-2 serology result using Student’s t-test for continuous variables and χ2 test for categorical variables; if expected counts were less than 5, we applied the Fisher’s exact test . We performed a multivariable logistic regression to evaluate characteristics associated with SARS-CoV-2 seropositivity. Adjustment variables were age, sex, work sector and all variables significantly associated in bivariable analyses, adopting a significance level of 0.1, with the exception of variables issued from the same branching logic, for which only the most pertinent was kept.

Missing data were excluded from the analyses. All statistical analyses were conducted using Stata, version 15.0. Statistical significance was set at a level of P <0.05.

**RESULTS**

Characteristics of the 455 essential workers included in the study are listed according to the work sector in Table A as Supplemental Digital Content, <http://links.lww.com/JOM/B191>. The average age was 44.3 years (SD 11.5) with the oldest group consisting of the bus drivers (47.1 years, SD 9.3). Female workers were predominant in food supermarkets (65.4%) and in the laundry services (77.9%) while only 16 (15.4%) bus drivers were women. Laundry employees adopted more protective behaviors against SARS-CoV-2, both in private life and at the workplace, than the other types of workers. For example, laundry employees implemented a higher use of masks in public places (33.7% vs 5.8% among bus-drivers, 3.0% in food supermarket employees and 7.9% among mail-sorting center workers) and at work (83.8% vs 4.0% among bus-drivers, 3.3% in food supermarket employees and 1.9% among mail-sorting center workers). Food supermarket workers reported more frequent close contact at the workplace with people having symptoms suggestive of SARS-CoV-2 infection (22.8% vs 14% in all employees) or with persons having tested positive for SARS-CoV-2 (17.1% vs 7.2%) (Table A as Supplemental Digital Content, <http://links.lww.com/JOM/B191>).

Among the 428 participants working on site, 68 tested positive for SARS-CoV-2 IgG and/or IgA (Table 2) and among them 37 (54.4%) reported flu-like symptoms since the end of February 2020 .The overall crude SARS-CoV-2 seroprevalence was 15.9% (95% CI 12.6 -19.7) (Table 2). The seroprevalence found in the local general population aged 20-64 years old at the same period was 11.2% (7.1-15.2). The seroprevalence in the selected essential workers was thus higher (RR= 1.44, 0.94-2.19) than that in the general population, but not significantly higher (P-value = 0.089). When each work sector was considered independently, only food supermarket workers had a statistically significant higher seroprevalence (22.0%, 15.0-30.3, P = 0.006). Notably, compared to the general population, the seroprevalence of workers in store 5 was more than three times higher (37.9%, 25.5-51.6, P <0.001). Regarding the other sectors, the seroprevalence was highest among employees of the mail-sorting service (16.2%, 9.7-24.7), followed by the laundry services’ employees (12.1%, 6.4-20.2) and the bus drivers (11.9%, 6.3-19.8).

No other statistically significant difference with the local general population aged 20-64 years was found stratifying by work function or workplace, including the four laundry workplaces (Table 2). The participants’ characteristics according to serology result are displayed in Table B as Supplemental Digital Content, <http://links.lww.com/JOM/B192>. In multivariable analysis, variables associated with seropositivity were being a food supermarket worker (adjusted Odds Ratio (aOR) 2.67, 95% CI 1.01-7.10, P 0.049), having experienced flu-like symptoms since the end of February 2020 (aOR 2.65, 1.43-4.88, P 0.002), having at least one housemate who tested positive by reverse transcriptase polymerase chain reaction (RT-PCR) (aOR 8.16, 1.41-47.08, P 0.019) and respecting hygiene rules in private life (aOR 3.81, 1.09-13.39, P 0.037) (Table 3).

**DISCUSSION**

This study investigated the SARS-CoV-2 seroprevalence during the first semi-lockdown in the Canton of Vaud, Switzerland, among four types of essential workers who are at theoretical moderate to high risk of exposure due to their close interactions with customers or colleagues and/or contact with potentially infected surfaces/material. The SARS-CoV-2 seroprevalence was overall higher (but not significantly) than that of local general working-age population. However, the seroprevalence varied according to the work sector, being similar among bus drivers, workers at the post service and at the laundries, but higher among food supermarket employees, compared to that of the general population. A history of flu-like symptoms, having a housemate with a confirmed COVID-19 infection, compliance with hygiene rules in private life, and being a food supermarket worker significantly increased the odds of testing positive for SARS-CoV-2 antibodies.

Consistent with previous findings (10, 15), the overall risk of SARS-CoV-2 infection among these four-types of essential workers was not significantly increased. These findings might be the result of the appropriate implementation of safety procedures at workplace. Two facts are in favor of this hypothesis. First, all four companies put in place, during the study period, a range of measures to protect their employees and customers (Table 1). Second, a lower seroprevalence was found among employees of the laundry, who better implemented protective measures, while the highest seropositivity was detected among food supermarket workers who reported a less adequate implementation of protective measures at work. Furthermore, the low seroprevalence among bus drivers could be partially attributed to the decreased use of public transport during the first wave.

Another hypothesis is that most workers got infected at home rather than at work, and therefore had the same risk of exposure as the general working age population. Indeed, consistent with results of previous research among essential workers (16, 32), having a housemate who tested positive for SARS-CoV-2 was associated with seropositivity in our study. Conversely, after controlling for potential confounding variables, having a close workplace contact with a person who tested positive for SARS-CoV-2 was not associated with seropositivity. This is consistent with similar results among health care workers showing that SARS-CoV-2 seroconversion was associated with household transmission, but not with working in a COVID-19 unit (32, 33). Moreover, previous research found a higher risk of infection from exposure to a household member than from other types of exposure (34, 35).

A higher seroprevalence than that of the general working age population was found for food supermarket workers. Specifically, this was the case for one of the five food stores, suggesting that exposure to SARS-CoV-2 may have occurred mostly in the workplace rather than at home for employees of this specific store (n°5). Taking into account that all food stores implemented the same kind of precautionary measures, we hypothesized two scenarios: 1) an outbreak occurred within store n°5 through contacts among colleagues, probably when they were eating close to each other and without masks during breaks; 2) an exposure to customers with a much higher incidence of COVID-19 than customers at other stores, which is supported by a high number of COVID-19 cases in the city of store n°5 during the first weeks of the epidemic (36). One could argue that the latter observation would support the hypothesis of workers having been infected at home rather than at work; however, many food workers of store n°5 were in fact living in other cities with lower transmission. Further detailed analyses in this subsample could not be performed due to the small number of store 5 participants (n=58).

Regarding mail-sorting employees, the overall seroprevalence was higher than that of the general working age population but not significantly. Interestingly, nearly a quarter of the people working in the main hall were positive, but no cases were found among office staff. This suggests that transmission within a large open space, where close contacts with many different collaborators cannot be avoided, might still have played a role. The finding that complying with hygiene rules in private life (ie. frequent hand washing, sneezing into the elbow, using disposable handkerchiefs) was associated with seropositivity was unexpected. It might be a chance finding, since an elevated compliance with the hygiene rules was observed in most of the participants (94.1% of seropositive vs 82.8% of seronegative workers). Finally, our finding that reporting a history of flu-like symptoms was associated with SARS-CoV-2 seropositivity is in line with other studies among essential workers (10, 37).

This study has some limitations. First, the participation rate was lower than expected, limiting the precision of the results. Second, participants with a history of COVID-19-like symptoms as well as those with a household or a work exposure were more likely to take part in the study, potentially leading to overestimate the seroprevalence. However, since this applies also to the local working-age population, it should not impact the difference in seroprevalence. Finally, the local general population sample aged 20-64 was small (n=235) and may include other essential workers at moderate-high occupational risk of exposure to coronavirus infection, which may lead to an underestimation of the difference in SARS-COV-2 seroprevalence between the study population and this comparison group.

One clear strength of this study is the extensive set of covariates collected and taken into account as potential confounders in multivariable analysis.

**CONCLUSIONS**

The overall SARS-CoV-2 seroprevalence among essential workers of four sectors was similar to that of the general working-age population during the first COVID-19 wave in the Canton of Vaud. The implementation of protective measures against SARS-CoV-2 infection at workplace could have mitigated the risk of exposure. Our results show that exposure in the workplace may have contributed to transmission among food-store workers. However, as shown in several studies looking at other types of workers, “at home” exposure seems to be the most probable source of infections among these essential workers, which is also suggested by the fact that the strongest predictor of seropositivity was having a housemate positive for SARS-CoV-2. Our results highlight the importance of combining work-specific protective measures with universal public health measures to be applied in private settings.

**ABBREVIATIONS**

aOR: adjusted odds ratio

BMI: body mass index

CI: confidence interval

COVID-19: coronavirus disease 2019

IgA: immunoglobulin A

IgG: immunoglobulin G

RT-PCR: reverse transcriptase-polymerase chain reaction

RR: Risk ratio

SARS-CoV-2: severe acute respiratory syndrome coronavirus 2

SD: standard deviation

WHO: World Health Organization

**REFERENCES**

1. Cucinotta D, Vanelli M. WHO Declares COVID-19 a Pandemic. Acta bio-medica : Atenei Parmensis. 2020;91(1):157-60.

2. Scire J, Nadeau, S., Vaughan, T. G., Brupbacher, G., Fuchs, S., Sommer, J., et al. (2020). Reproductive number of the COVID-19 epidemic in Switzerland with a focus on the Cantons of Basel- Stadt and Basel- Landschaft. Swiss Med. Wkly. 150, 20271. doi: 10.4414/smw.2020.20271.

3. Our world data (2020). Switzerland: Coronavirus Pandemic Country Profile. Available online at: <https://ourworldindata.org/coronavirus/country/switzerland?country=C>̃HE (accessed October 16, 2020). [

4. Riguzzi M, Gashi S. Lessons From the First Wave of COVID-19: Work-Related Consequences, Clinical Knowledge, Emotional Distress, and Safety-Conscious Behavior in Healthcare Workers in Switzerland. Frontiers in psychology. 2021;12:628033.

5. The L. The plight of essential workers during the COVID-19 pandemic. Lancet (London, England). 2020;395(10237):1587.

6. Gaitens J, Condon M, Fernandes E, McDiarmid M. COVID-19 and Essential Workers: A Narrative Review of Health Outcomes and Moral Injury. International journal of environmental research and public health. 2021;18(4).

7. <https://www.publicsafety.gc.ca/cnt/ntnl-scrt/crtcl-nfrstrctr/esf-sfe-en.aspx> [

8. <https://www.cdc.gov/vaccines/covid-19/categories-essential-workers.html> [

9. <https://www.ilo.org/legacy/english/dialogue/ifpdial/llg/noframes/ch5.htm#6> [

10. Poustchi H, Darvishian M, Mohammadi Z, Shayanrad A, Delavari A, Bahadorimonfared A, et al. SARS-CoV-2 antibody seroprevalence in the general population and high-risk occupational groups across 18 cities in Iran: a population-based cross-sectional study. The Lancet Infectious diseases. 2021;21(4):473-81.

11. Chughtai OR, Batool H, Khan MD, Chughtai AS. Frequency of COVID-19 IgG Antibodies among Special Police Squad Lahore, Pakistan. Journal of the College of Physicians and Surgeons--Pakistan : JCPSP. 2020;30(7):735-9.

12. Sabourin KR, Schultz J, Romero J, Lamb MM, Larremore D, Morrison TE, et al. Risk Factors of SARS-CoV-2 Antibodies in Arapahoe County First Responders-The COVID-19 Arapahoe SErosurveillance Study (CASES) Project. Journal of occupational and environmental medicine. 2021;63(3):191-8.

13. West EA, Anker D, Amati R, Richard A, Wisniak A, Butty A, et al. Corona Immunitas: study protocol of a nationwide program of SARS-CoV-2 seroprevalence and seroepidemiologic studies in Switzerland. International journal of public health. 2020;65(9):1529-48.

14. Winter AK, Hegde ST. The important role of serology for COVID-19 control. The Lancet Infectious diseases. 2020;20(7):758-9.

15. Stringhini S, Zaballa ME, Pullen N, de Mestral C, Perez-Saez J, Dumont R, et al. Large variation in anti-SARS-CoV-2 antibody prevalence among essential workers in Geneva, Switzerland. Nature communications. 2021;12(1):3455.

16. Sami S, Akinbami LJ, Petersen LR, Crawley A, Lukacs SL, Weiss D, et al. Prevalence of SARS-CoV-2 Antibodies in First Responders and Public Safety Personnel, New York City, New York, USA, May-July 2020. Emerging infectious diseases. 2021;27(3):796-804.

17. Shukla V, Lau CSM, Towns M, Mayer J, Kalkbrenner K, Beuerlein S, et al. COVID-19 Exposure Among First Responders in Arizona. Journal of occupational and environmental medicine. 2020;62(12):981-5.

18. Pasqualotto AC, Pereira PC, Lana DFD, Schwarzbold AV, Ribeiro MS, Riche CVW, et al. COVID-19 seroprevalence in military police force, Southern Brazil. PloS one. 2021;16(4):e0249672.

19. McGuire SS, Klassen AB, Heywood J, Sztajnkrycer MD. Prevalence of COVID-19 IgG Antibodies in a Cohort of Municipal First Responders. Prehospital and disaster medicine. 2021;36(2):131-4.

20. Zaidi S, Rizwan F, Riaz Q, Siddiqui A, Khawaja S, Imam M, et al. Seroprevalence of anti-SARS-CoV-2 antibodies in residents of Karachi-challenges in acquiring herd immunity for COVID 19. Journal of public health (Oxford, England). 2020.

21. Bui DP, McCaffrey K, Friedrichs M, LaCross N, Lewis NM, Sage K, et al. Racial and Ethnic Disparities Among COVID-19 Cases in Workplace Outbreaks by Industry Sector - Utah, March 6-June 5, 2020. MMWR Morbidity and mortality weekly report. 2020;69(33):1133-8.

22. Günther T, Czech-Sioli M, Indenbirken D, Robitaille A, Tenhaken P, Exner M, et al. SARS-CoV-2 outbreak investigation in a German meat processing plant. EMBO molecular medicine. 2020:e13296.

23. Waltenburg MA, Victoroff T, Rose CE, Butterfield M, Jervis RH, Fedak KM, et al. Update: COVID-19 Among Workers in Meat and Poultry Processing Facilities - United States, April-May 2020. MMWR Morbidity and mortality weekly report. 2020;69(27):887-92.

24. Park SY, Kim YM, Yi S, Lee S, Na BJ, Kim CB, et al. Coronavirus Disease Outbreak in Call Center, South Korea. Emerging infectious diseases. 2020;26(8):1666-70.

25. Hossain A, Nasrullah SM, Tasnim Z, Hasan MK, Hasan MM. Seroprevalence of SARS-CoV-2 IgG antibodies among health care workers prior to vaccine administration in Europe, the USA and East Asia: A systematic review and meta-analysis. EClinicalMedicine. 2021;33:100770.

26. Galanis P, Vraka I, Fragkou D, Bilali A, Kaitelidou D. Seroprevalence of SARS-CoV-2 antibodies and associated factors in healthcare workers: a systematic review and meta-analysis. The Journal of hospital infection. 2021;108:120-34.

27. Alali WQ, Bastaki H, Longenecker JC, Aljunid SM, AlSeaidan M, Chehadeh W, et al. Seroprevalence of SARS-CoV-2 in migrant workers in Kuwait. Journal of travel medicine. 2021;28(2).

28. Gujski M, Jankowski M, Pinkas J, Wierzba W, Samel-Kowalik P, Zaczyński A, et al. Prevalence of Current and Past SARS-CoV-2 Infections among Police Employees in Poland, June-July 2020. Journal of clinical medicine. 2020;9(10).

29. Addetia A, Crawford KH, Dingens A, Zhu H, Roychoudhury P, Huang ML, et al. Neutralizing antibodies correlate with protection from SARS-CoV-2 in humans during a fishery vessel outbreak with high attack rate. medRxiv. 2020.

30. <https://www.who.int/news-room/q-a-detail/coronavirus-disease-covid-19-health-and-safety-in-the-workplace>. [

31. Fenwick C, Croxatto A, Coste AT, Pojer F, André C, Pellaton C, et al. Changes in SARS-CoV-2 Spike versus Nucleoprotein Antibody Responses Impact the Estimates of Infections in Population-Based Seroprevalence Studies. Journal of virology. 2021;95(3).

32. Meylan S, Dafni U, Lamoth F, Tsourti Z, Lobritz MA, Regina J, et al. SARS-CoV-2 seroprevalence in healthcare workers of a Swiss tertiary care centre at the end of the first wave: a cross-sectional study. BMJ open. 2021;11(7):e049232.

33. Steensels D, Oris E, Coninx L, Nuyens D, Delforge ML, Vermeersch P, et al. Hospital-Wide SARS-CoV-2 Antibody Screening in 3056 Staff in a Tertiary Center in Belgium. Jama. 2020;324(2):195-7.

34. Bi Q, Lessler J, Eckerle I, Lauer SA, Kaiser L, Vuilleumier N, et al. Insights into household transmission of SARS-CoV-2 from a population-based serological survey. Nature communications. 2021;12(1):3643.

35. Dupraz J, Butty A, Duperrex O, Estoppey S, Faivre V, Thabard J, et al. Prevalence of SARS-CoV-2 in household members and other close contacts of COVID-19 cases: a serologic study in canton of Vaud, Switzerland. Open Forum Infectious Diseases. 2021.

36. Oral communication from the Office of the Chief medical officier of Canton of Vaud to VDA.

37. Mulchandani R, Taylor-Philips S, Jones HE, Ades AE, Borrow R, Linley E, et al. Association between self-reported signs and symptoms and SARS-CoV-2 antibody detection in UK key workers. The Journal of infection. 2021;82(5):151-61.

**FIGURES LIST**

**Figure 1. Flow chart of participants**

**SUPPLEMENTAL DIGITAL CONTENTS LIST**

Table A. Participants’ characteristic according to their work sector

Table B. Characteristics of participants working on site according to SARS-CoV-2 serology result
