# Supplementary material for: SARS-CoV-2 Seroprevalence in Employees of Four Essential Non–Health Care Sectors at Moderate/High Risk of Exposure to Coronavirus Infection: Data From the “First Wave”
Source: J Occup Environ Med. 2022 Sep 9;65(1):10–5. doi: 10.1097/JOM.0000000000002690 (PMC9835238; doi:10.1097/JOM.0000000000002690)
Supplement: Supplementary file 2 [file joem-65-010-s002.docx]

**Table B. Characteristics of participants working on site according to SARS-CoV-2 serology result**

|  | **Participants working on site n=428** | **SARS-CoV-2 positivity** | | |
| --- | --- | --- | --- | --- |
|  |  | **No**  **(n=360)** | **Yes**  **(n=68)** | **P *** |
| Age, y, mean±SD | 44.0±11.6 | 43.8 ± 11.9 | 44.9 ± 9.4 | .451 |
| Company |  |  |  | .130 |
| - Food supermarket | 123 (28.7) | 96 (26.7) | 27 (39.7) |  |
| - Public transportation | 101 (23.6) | 89 (24.7) | 12 (17.7) |  |
| - Mail-sorting service | 105 (24.5) | 88 (24.4) | 17 (25.0) |  |
| - Laundry | 99 (23.1) | 87 (24.2) | 12 (17.7) |  |
| Women | 215 (50.2) | 183 (38.3) | 32 (47.1) | .554 |
| Education |  |  |  | .877 |
| - No or low education | 165 (38.6) | 141 (39.1) | 24 (35.3) |  |
| - Upper secondary education | 201 (47.0) | 168 (46.7) | 33 (48.5) |  |
| - Tertiary education | 55 (12.9) | 46 (12.8) | 9 (13.2) |  |
| Comorbidity (≥1 chronic diseases)† | 86 (20.1) | 72 (20.0) | 14 (20.6) | .912 |
| Current smoker | 119 (27.8) | 105 (29.2) | 14 (20.6) | .148 |
| BMI |  |  |  | .339 |
| - Under/normal weight (BMI<25) | 192 (44.9) | 156 (43.3) | 36 (52.9) |  |
| - Overweight (25≤BMI<30) | 159 (37.2) | 137 (38.1) | 22 (32.4) |  |
| - Obesity (BMI≥30) | 77 (18.0) | 67 (18.6) | 10 (14.7) |  |
| Flu-like symptoms since the end of February 2020 | 135 (31.5) | 98 (27.2) | 37 (54.4) | **<.001** |
| Having at least 1 housemate with symptoms suggestive of COVID-19^‡^ since the end of February 2020 | 52 (12.1) | 39 (10.8) | 13 (19.1) | .055 |
| Having at least 1 housemate tested RT-PCR positive | 8 (1.9) | 2 (0.6) | 6 (8.8) | **<.001** |
| Respect of hygiene rules in private life^§^ | 362 (84.6) | 298 (82.8) | 64 (94.1) | **.009** |
| Respect of distancing rules in private life^‖^ | 259 (60.5) | 215 (59.7) | 44 (64.7) | .472 |
| Meeting of 1 or more people/week, other than the housemates, during the semi-lockdown | 388 (90.7) | 327 (90.8) | 61 (87.7) | .612 |
| Public transport as main means of transport during the semi-lockdown | 59 (13.8) | 48 (13.3) | 11 (16.2) | .539 |
| Wearing always a mask in public places | 46 (10.7) | 34 (9.4) | 12 (17.7) | **.040** |
| At least 1 travel abroad since the end of February 2020 | 43 (10.0) | 34 (9.4) | 9 (13.2) | .299 |
| At least 1 close contact with people, other than the housemates, having symptoms suggestive of COVID-19^‡^, from 24 hours before symptoms onset | 57 (13.3) | 43 (11.9) | 14 (20.6) | **.045** |
| Change in one’s working conditions since SARS-CoV-2 outbreak | 207 (48.4) | 166 (46.1) | 41 (60.3) | **.032** |
| - Decreased working activity | 74 (17.3) | 65 (18.1) | 9 (13.2) | .335 |
| - Stop working | 4 (0.9) | 3 (0.8) | 1 (1.5) | .501 |
| - Teleworking | 21 (4.9) | 18 (5.0) | 3 (4.4) | 1.000 |
| - Stop because of a disease | 23 (5.4) | 13 (3.6) | 10 (14.7) | **<.001** |
| - Stop because of unemployment | 3 (0.7) | 2 (0.6) | 1 (1.5) | .406 |
| Work activity rate |  |  |  | .440 |
| - 75-100% | 286 (66.8) | 236 (65.6) | 50 (73.5) |  |
| - 50-74% | 110 (25.7) | 96 (26.7) | 14 (20.6) |  |
| - <50% | 32 (7.5) | 28 (7.8) | 4 (5.9) |  |
| At least 1 close contact at work with people having symptoms suggestive of COVID-19^‡^, from 24h before the symptoms outbreak | 60 (14.0) | 47 (13.1) | 13 (19.1) | .190 |
| At least 1 close contact at work with a person tested positive for SARS-CoV-2, from 24h before the symptoms outbreak | 31 (7.2) | 19 (5.3) | 12 (17.6) | <.001 |
| Carpooling to go to work | 55 (12.9) | 46 (12.8) | 9 (13.2) | .918 |
| Implementation of hygiene measures at work | 307 (71.7) | 254 (70.6) | 53 (77.9) | .239 |
| Implementation of social distancing at work | 183 (42.8) | 148 (41.1) | 35 (51.5) | .126 |
| Adequate implementation of protective measures at work | 213 (49.8) | 178 (49.4) | 35 (51.5) | .621 |
| Wearing a mask most of the time at work | 93 (21.7) | 80 (22.2) | 13 (19.1) | .555 |
| Hand sanitizer availability at work | 423 (98.8) | 355 (98.6) | 68 (100) | - |
| Mask availability at work | 383 (89.5) | 320 (88.9) | 63 (92.6) | .242 |
| Physical barriers availability at work | 227 (53.0) | 186 (51.7) | 41 (60.4) | .178 |
| Adequate information on protective measures at work | 334 (78.0) | 281 (78.1) | 53 (77.9) | .952 |

Note: unless otherwise specified data are displayed as n(%)

* From Student’s t-test for continuous variables and χ2 test or Fisher exact test, where appropriate, for categorical variables.

† Defined as presenting at least one chronic disease among hypertension, diabetes, cardiovascular disease, renal disease, chronic respiratory disease, immune weakness, active cancer and other chronic diseases.

‡ Defined as presenting cough or sore throat or shortness of breath or fever or fatigue or muscle pain or loss of smell or taste.

^§^Defined as frequent hand washing, sneezing into the elbow, use of disposable handkerchiefs etc.

^‖^Defined as physical distancing, avoid shaking hands or kissing, staying home, avoiding getting out if unnecessary.
