# Supplementary material for: SARS-CoV-2 Seroprevalence in Employees of Four Essential Non–Health Care Sectors at Moderate/High Risk of Exposure to Coronavirus Infection: Data From the “First Wave”
Source: J Occup Environ Med. 2022 Sep 9;65(1):10–5. doi: 10.1097/JOM.0000000000002690 (PMC9835238; doi:10.1097/JOM.0000000000002690)
Supplement: Supplementary file 3 [file joem-65-010-s003.docx]

**Table A. Participants’ characteristic according to their work sector**

|  | **Total population**  **n=455** | **Food supermarket n=133** | **Transportation**  **n=104** | **Mail-sorting service**  **n=114** | **Laundry**  **n=104** |
| --- | --- | --- | --- | --- | --- |
| Age, y, mean±SD | 44.3±11.5 | 40.3 ± 12.8 | 47.1 ± 9.3 | 46.6 ± 11.6 | 44.0 ± 10.5 |
| Women | 233 (51.2) | 87 (65.4) | 16 (15.4) | 49 (43.0) | 81 (77.9) |
| Education |  |  |  |  |  |
| - No or low education | 175 (38.5) | 45 (33.8) | 36 (34.6) | 39 (34.2) | 55 (52.9) |
| - Upper secondary education | 215 (47.3) | 69 (51.9) | 56 (53.8) | 56 (49.1) | 34 (32.7) |
| - Tertiary education | 57 (12.5) | 18 (13.5) | 11 (10.6) | 16 (14.0) | 12 (11.5) |
| Comorbidity (≥1 chronic diseases)* | 105 (23.1) | 25 (18.8) | 22 (21.2) | 37 (32.5) | 21 (20.2) |
| Current smoker | 125 (27.5) | 36 (27.1) | 27 (26.0) | 28 (24.6) | 34 (32.7) |
| BMI |  |  |  |  |  |
| - Under/normal weight (BMI<25) | 202 (44.4) | 73 (54.9) | 37 (35.6) | 51 (44.7) | 41 (39.4) |
| - Overweight (25≤BMI<30) | 170 (37.4) | 35 (26.3) | 50 (48.1) | 48 (42.1) | 37 (35.6) |
| - Obesity (BMI≥30) | 83 (18.2) | 25 (18.8) | 17 (16.3) | 15 (13.2) | 26 (25.0) |
| Flu-like symptoms since the end of February 2020 | 147 (32.3) | 50 (35.6) | 36 (34.6) | 36 (31.6) | 25 (24.0) |
| History of a positive RT-PCR test | 6 (1.3) | 5 (3.8) | 0 | 1 (0.9) | 0 |
| Having at least 1 housemate with symptoms suggestive of COVID-19† since the end of February 2020 | 60 (13.2) | 19 (14.3) | 13 (12.5) | 9 (7.9) | 19 (18.3) |
| Having at least 1 housemate tested RT-PCR positive | 13 (2.9) | 7 (5.3) | 3 (2.9) | 1 (0.9) | 2 (1.9) |
| Respect of hygiene rules in private life^‡^ | 382 (84.0) | 110 (82.7) | 88 (84.6) | 90 (79.0) | 94 (90.4) |
| Respect of distancing rules in private life^§^ | 276 (60.7) | 73 (54.9) | 65 (62.5) | 72 (63.2) | 66 (63.5) |
| Meeting of 1 or more people/week, other than the housemates, during the semi-lockdown | 403 (88.6) | 121 (91.0) | 94 (90.4) | 97 (85.1) | 91 (87.5) |
| Public transport as main means of transport during the semi-lockdown | 59 (13.0) | 21 (15.8) | 22 (21.2) | 1 (0.9) | 15 (14.4) |
| Wearing always a mask in public places | 54 (11.9) | 4 (3.0) | 6 (5.8) | 9 (7.9) | 35 (33.7) |
| At least 1 travel abroad since the end of February 2020 | 44 (9.7) | 19 (14.3) | 16 (15.4) | 6 (5.3) | 3 (2.9) |
| At least 1 close contact with people, other than the housemates, having symptoms suggestive of COVID-19†, from 24 hours before symptoms onset | 61 (13.4) | 28 (21.1) | 5 (4.8) | 18 (15.8) | 10 (9.6) |
| Change in one’s working conditions since SARS-CoV-2 outbreak | 229 (50.3) | 62 (46.6) | 66 (63.5) | 57 (50.0) | 44 (42.3) |
| - Decreased working activity | 76 (16.7) | 12 (9.0) | 44 (42.3) | 12 (10.5) | 8 (7.7) |
| - Stop working | 12 (2.6) | 5 (3.8) | 1 (1.0) | 4 (3.5) | 2 (1.9) |
| - Teleworking | 22 (4.8) | 2 (1.5) | 0 | 17 (14.9) | 3 (2.9) |
| - Stop because of a disease | 32 (7.0) | 12 (9.0) | 9 (8.7) | 6 (5.3) | 5 (4.8) |
| - Stop because of unemployment | 3 (0.7) | 0 | 3 (2.9) | 0 | 0 |
| Going to the workplace, partially or fully, during semi-lockdown | 428 (94.1) | 123 (92.5) | 101 (97.1) | 105 (92.1) | 99 (95.2) |
| **EXPOSURE AT WORK FOR THOSE WHO, PARTIALLY OR FULLY, WORKED ON SITE** | **N=428** | **N=123** | **N=101** | **N=105** | **N=99** |
| Work activity rate |  |  |  |  |  |
| - 75-100% | 286 (66.8) | 63 (51.2) | 70 (69.3) | 73 (69.5) | 80 (80.1) |
| - 50-74% | 110 (25.7) | 42 (34.1) | 28 (27.8) | 28 (26.7) | 12 (12.1) |
| - <50% | 32 (7.5) | 18 (14.6) | 3 (3.0) | 4 (3.8) | 7 (7.1) |
| At least 1 close contact with people having symptoms suggestive of COVID-19† at workplace, from 24h before the symptoms outbreak | 60 (14.0) | 28 (22.8) | 4 (4.0) | 16 (15.2) | 12 (12.1) |
| At least 1 close contact at work with a person tested positive for SARS-CoV-2, from 24h before the symptoms outbreak | 31 (7.2) | 21 (17.1) | 0 (0.0) | 6 (5.7) | 4 (4.0) |
| Carpooling to go to work | 55 (12.9) | 11 (8.9) | 12 (11.9) | 10 (9.5) | 22 (22.2) |
| Implementation of hygiene measures at work | 307 (72.1) | 82 (66.7) | 60 (59.4) | 72 (68.6) | 93 (93.9) |
| Implementation of social distancing at work | 183 (42.8) | 29 (23.6) | 48 (47.5) | 51 (48.6) | 55 (55.6) |
| Adequate implementation of protective measures at work | 213 (49.8) | 34 (27.6) | 51 (50.5) | 50 (47.6) | 78 (78.8) |
| Wearing a mask most of the time at work | 93 (21.7) | 4 (3.3) | 4 (4.0) | 2 (1.9) | 83 (83.8) |
| Hand sanitizer availability at work | 423 (98.8) | 121 (98.4) | 99 (98.0) | 105 (100.0) | 98 (99.0) |
| Mask availability at work | 383 (89.5) | 105 (85.4) | 88 (87.1) | 91 (86.7) | 99 (100) |
| Physical barriers availability at work | 227 (53.0) | 103 (83.7) | 78 (77.2) | 40 (38.1) | 6 (6.1) |
| Adequate information on protective measures at work | 334 (78.0) | 90 (73.2) | 68 (67.3) | 87 (82.9) | 89 (90.0) |

Note: unless otherwise specified data are displayed as n(%)

*Defined as presenting at least one chronic disease among hypertension, diabetes, cardiovascular disease, renal disease, chronic respiratory disease, immune weakness, active cancer and other chronic diseases.

† Defined as presenting cough or sore throat or shortness of breath or fever or fatigue or muscle pain or loss of smell or taste

‡Defined as frequent hand washing, sneezing into the elbow, use of disposable handkerchiefs etc.

^§^ Defined as physical distancing, avoid shaking hands or kissing, staying home, avoiding getting out if unnecessary.
